# Supplementary material for: The interactome of intact mitochondria by cross-linking mass spectrometry provides evidence for coexisting respiratory supercomplexes
Source: Mol Cell Proteomics. 2017 Dec 8;17(2):216–32. doi: 10.1074/mcp.RA117.000470 (PMC5795388; doi:10.1074/mcp.RA117.000470)
Supplement: Supplemental Data [file supp_RA117.000470_133922_0_supp_23978_fzffwf.zip › spectra_annotation/mito_DR_spectra_annotation/23-1-7-1-9-1.pdf]

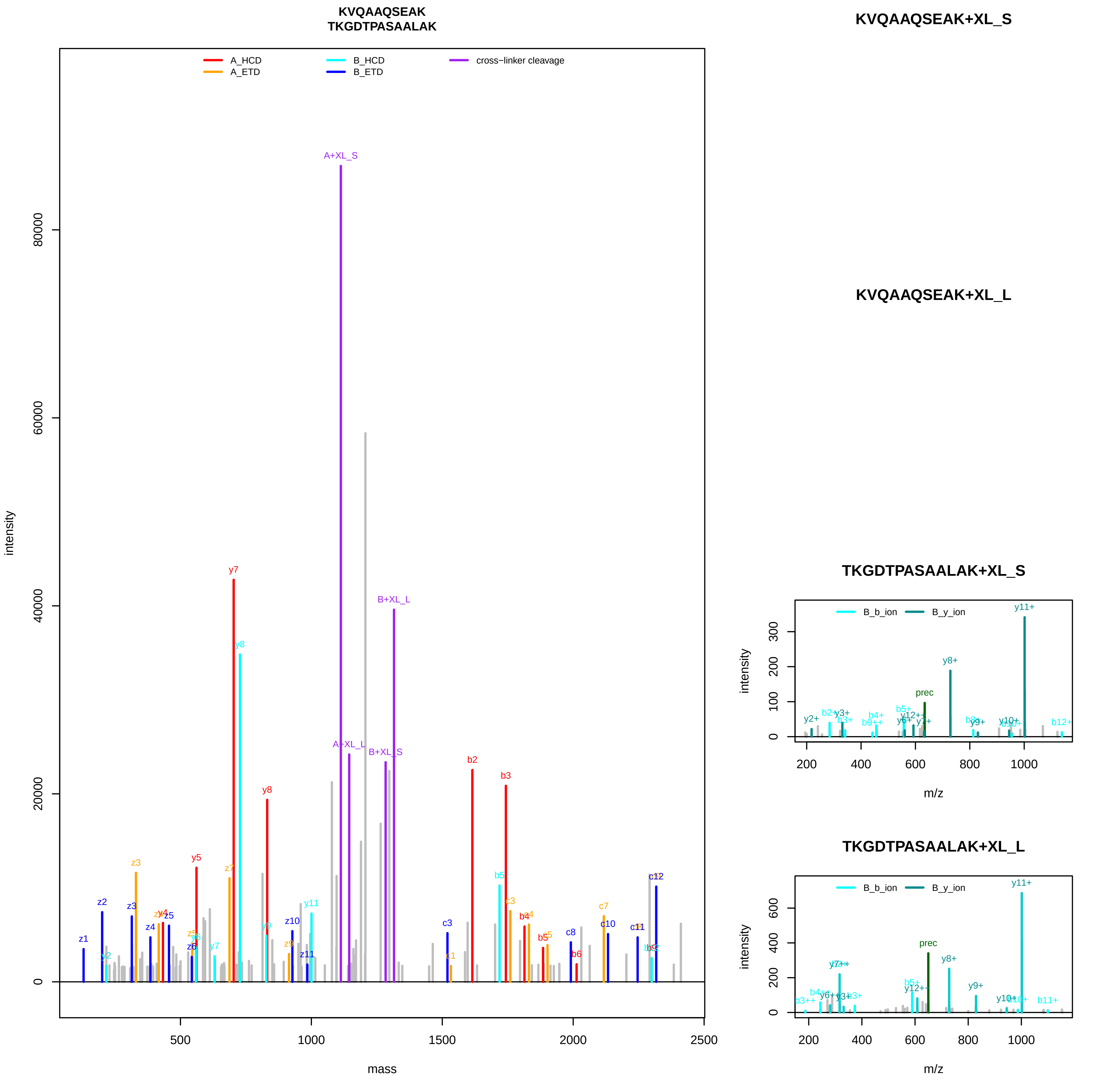
KVQAAQSEAK
TKGDTPASAALAK
A_HCD
A_ETD
B_HCD
B_ETD
cross−linker cleavage
A+XL_S
B+XL_L
A+XL_L
B+XL_S
y7
y8
y5
z3
c12
b2
b3
b5
c3
c7
c10
c11
c8
b4
b6
z1
z2
z4
z5
z6
z10
z11
y11
y9
y2
y4
z7
z9
c1
intensity
mass
0
20000
40000
60000
80000
500
1000
1500
2000
2500
KVQAAQSEAK+XL_S
KVQAAQSEAK+XL_L
TKGDTPASAALAK+XL_S
B_b_ion
B_y_ion
y11+
y8+
prec
y2+
y3+
b12+
y10+
y9+
intensity
m/z
0
100
200
300
200
400
600
800
1000
TKGDTPASAALAK+XL_L
B_b_ion
B_y_ion
y11+
y8+
prec
y9+
b11+
intensity
m/z
0
200
400
600
200
400
600
800
1000
